# Water buffalo farming, udder health and its dairy production status in Bangladesh: Practices, challenges, and potentialities

**DOI:** 10.1007/s11259-025-10823-8

**Published:** 2025-08-28

**Authors:** Tishita Sen Ape, Shuvo Singha, Unusing Marma, Hasnat Jahan Rumi, Sirajul Islam Sagor, Antonella Chiariotti, Vittoria Lucia Barile, Ylva Persson, Md. Mizanur Rahman

**Affiliations:** 1 Department of Medicine and Surgery, Chattogram Veterinary and Animal Sciences University, Chattogram, Bangladesh; 2 Udder Health Bangladesh, Chattogram, Bangladesh; 3 Research Center for Animal Production and Aquaculture, Council for Agricultural Research and Economics (CREA), Monterotondo, Italy; 4 Department of Clinical Sciences, The Swedish University of Agricultural Sciences, Uppsala, Sweden; 5 Swedish Veterinary Agency, Uppsala, Sweden

**Keywords:** Milk value chain, Dairy chain constraint, Buffalo research, Sustainability

## Abstract

Buffalo farming in Bangladesh plays a key role in enhancing food security by providing a valuable protein source through milk and meat production. Water buffalo are well-adapted to harsh climate conditions due to their robust immune systems, while they may adapt with minimal feed availability compared to dairy cows. Despite its adaptability and importance, buffalo farming has been underprioritized compared to cattle farming due to several challenges, such as a lack of awareness among farmers of modern dairy practices, limited access to good quality feed, poor breeding programs, improper management practices, and inadequate veterinary services. Furthermore, the lack of a dedicated buffalo product chain, fragmented market, and limited policy support have further impeded the development of this sector, leading to reduced profitability. Despite similar climatic conditions, Bangladesh’s buffalo population remains underexploited, contributing minimally to global buffalo milk production. In contrast, several neighboring countries, including India, Pakistan, and Nepal, lead global buffalo milk production, highlighting a significant gap in Bangladesh’s buffalo sector. Despite these constraints, buffalo farming holds great potential, particularly for producing high-nutrient products such as yogurt and cheese, which are popular in many regions. Recognizing the potential of buffalo farming, the government and other organizations are collaborating on initiatives such as enhanced breeding programs, policy support, and extension services for farmers. This review highlights the challenges and prospects of water buffalo farming, including the need for strategic interventions and key research areas to exploit the potential in dairy enterprises in Bangladesh.

**Supplementary Information:**

The online version contains supplementary material available at 10.1007/s11259-025-10823-8.

## Introduction

Bangladesh’s agro-based economy largely relies on agriculture and livestock production. Among livestock species, the water buffalo plays a key role in food security after cattle, due to their ability to thrive in harsh environments and convert poor-quality forage into nutritious meat and high-fat milk (Cruz 2010; Kiran and Naveena 2014). However, in Bangladesh, the potential of water buffalo has not been fully exploited for milk production, as only 0.04% of global milk production comes from water buffalo (Chakravarty 2013).

Geographically, buffalo farming is concentrated in coastal areas, river basins, islands, and shoals, where buffalo adapt well to harsh climatic conditions including tidal waves, natural calamities, saline water, and low-input systems. Despite this, water buffalo has remained underprioritized compared to other livestock species and faces several challenges such as poor breeding, limited access to quality feed, lack of modern management practices, and insufficient veterinary support. Furthermore, a lack of a dedicated buffalo product chain and limited policy focus have constrained its development. Recently, buffalo farming has been increasingly supported by government and non-government organizations through improved breeding programs, targeted policy support, enhanced extension services, and farmer training programs, which aim to address the constraints and unlock the full potential of buffalo farming. This review sheds light on the present status, constraints, and prospects of water buffalo farming and production in Bangladesh, emphasizing the need for further research to identify strategic interventions.

### Systematic literature search

**Fig. 1:**
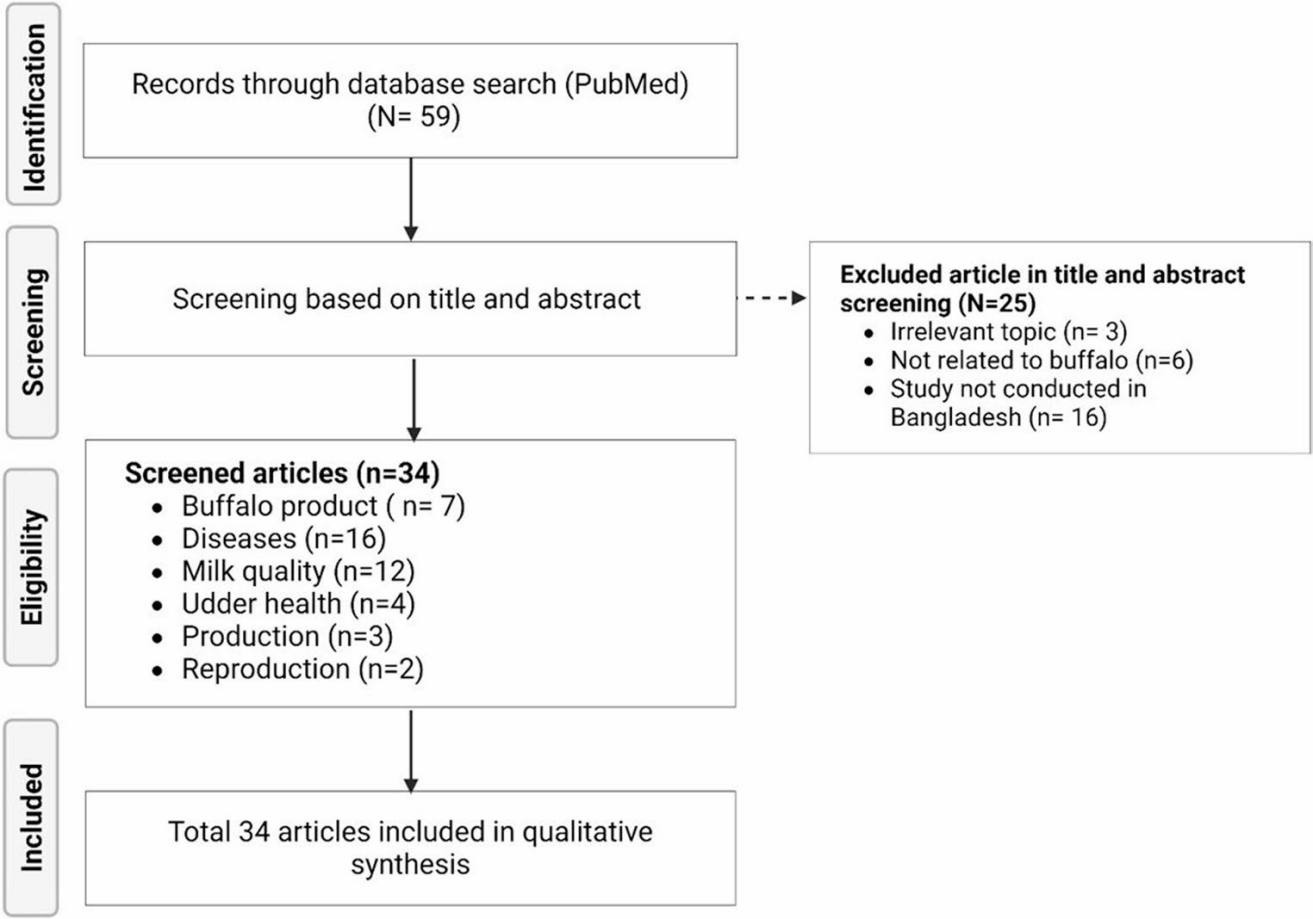
Flow diagram of 34 eligible studies selected for a systematic review on the potential, challenges, and prospects of water buffalo farming

We performed a systematic literature search in PubMed to identify publications reporting on buffalo farming and its dairy production status in Bangladesh, following the PRISMA guidelines (Page et al. 2021). A comprehensive search strategy was employed to identify the maximum number of relevant publications. Two different types of search terms were considered, based on the presence of these terms in the Title/Abstract. The keywords “buffalo” OR “bubaline” were used to identify the subject of interest, while “Bangladesh” was used to specify the geographic focus. No language or time restrictions were applied. The database search was conducted on March 1, 2025, and aimed to encompass all relevant studies on water buffalo in Bangladesh. A total of 59 articles published between 1985 and 2025 were identified in the initial search. Among these, 34 full-text articles were identified as eligible based on title and abstract screen and categorized based on the primary focus: buffalo products (*n* = 7), diseases (*n* = 16), milk quality (*n* = 2), udder health (*n* = 4), production (*n* = 3), and reproduction (*n* = 2) (Fig. 1). The remaining 25 articles were excluded due to irrelevant topics (*n* = 3), not related to buffalo (*n* = 6), and study not conducted in Bangladesh (*n* = 16) (Supplementary File 1). Notably, the majority of the included studies (*n* = 22; 65%) were published between 2024 and 2025 or are ongoing, indicating a recent surge of interest and prioritization in buffalo research in Bangladesh. The analysis of the existing literature indicates that research on buffalo in Bangladesh is limited, with many important areas remaining unexplored. However, a recent surge of interest and increased prioritization of buffalo research is evident. There is a pressing need for comprehensive, high-quality research focusing on various aspects of buffalo health, production, and management.

### Buffalo population and production

**Fig. 2:**
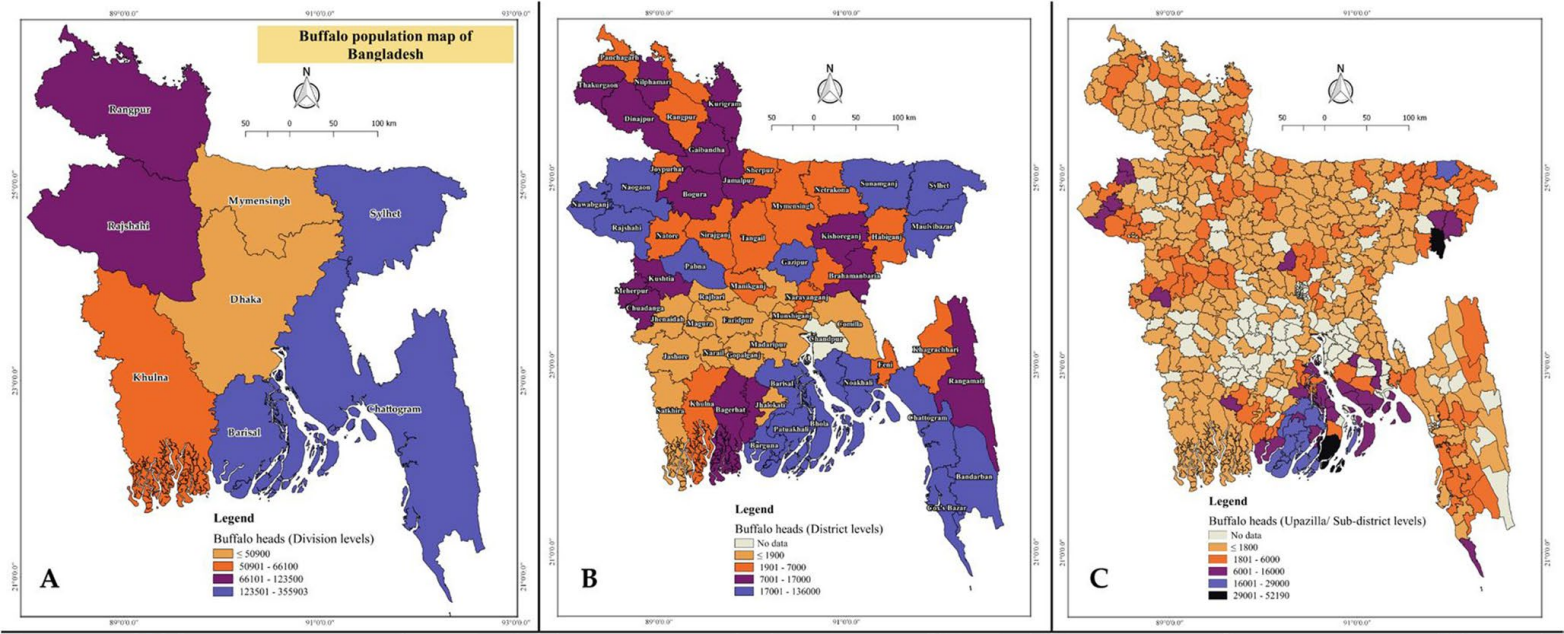
National and sub-national level buffalo concentration in Bangladesh. **a**) Eight divisions of BD **b**) 64 districts of BD **c**) 495 upazila of BD **(**LDDP 2019)

**Fig. 3:**
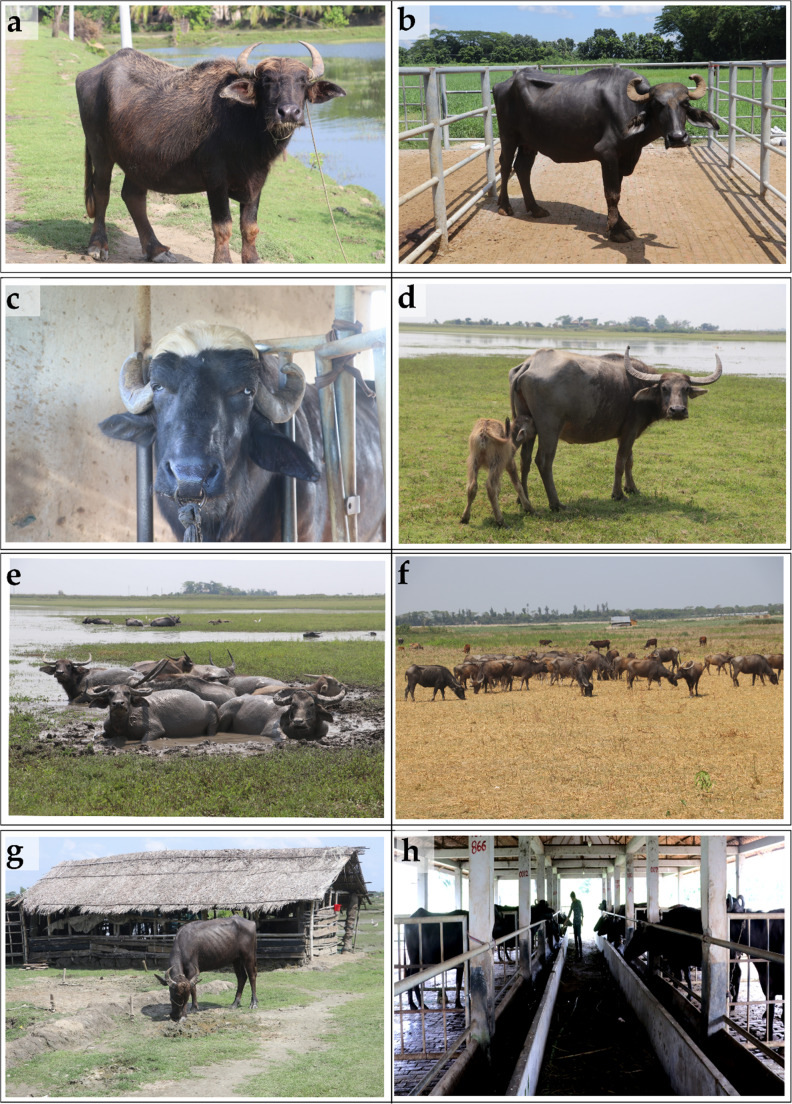
Available breeds and different types of buffalo (**a**-**d**) and rearing systems (**e**-**h**) in Bangladesh. (**a**) Indigenous non-descriptive breed (**b**) Murrah (**c**) Nili Ravi (**d**) Swamp type buffalo (**e**) Free-range or bathan (**f**) Semi-free-range or semi-bathan (**g**) Household (**h**) Intensive (Photo Source: Collected from Udder Health Bangladesh)

Buffalo population is increasing in a constant trend, particularly since 1998, in Asian countries than the rest of the world (Minervino et al. 2020) due to its easy adaptability in coastal areas, disease resilience, and utilization of minimal feed sources available (Terramoccia et al. 2000). As of 2022, the global buffalo population was approximately 205.1 million, with over 98% concentrated in Asia. In contrast, Europe, Africa, South America, and accounted for 0.2%, 0.7%, 1%, respectively. Among Asian countries, India has the largest buffalo population with 111.9 million buffaloes, producing an estimated 90 million tonnes of buffalo milk annually and contributing nearly 74% of global production. Pakistan follows with 43.7 million buffaloes, producing 37 million tonnes of buffalo milk per year, accounting for about 13% of global output. China, with 26.9 million buffaloes, contributes around 3% of global production, yielding approximately 3 million tonnes of milk. Nepal, with 5.1 million buffaloes, produces 1.46 million tonnes, contributing roughly 0.5% to the world’s buffalo milk supply. According to the 2023–24 data from the Department of Livestock Services, the buffalo population in Bangladesh is approximately 1.5 million, contributing less than 3% of the national and 0.04% of the global milk production (DLS 2023; FAOSTAT 2023). Despite having similar climate conditions (averaged temperature ranged 24–32^º^C; humidity ranged 55-85%) (Sharif and Dey 2021), buffalo population in Bangladesh is comparatively lower than the neighboring Southeast Asian countries. This might be due to the absence of a high milk-yielding buffalo breed, a lack of an appropriate breeding and development plan, inadequate policy support, and the lesser popularity of buffalo products. Based on the availability of feed resources and environmental conditions, the distribution of buffalo population varies significantly across regions (Fig. 2). Coastal districts, such as Bhola, Noakhali, Cox’s Bazar, and Barisal, have the highest concentrations of buffalo (BBS 2022). These areas provide non-competitive grazing lands compared to other dairy species due to increased salinity and contribute to mitigating seasonal feed shortages, thereby ensuring their survival throughout the year. In riverine areas, including the shoals (haor in Bengali) and floodplains of districts in Sylhet, Mymensingh, and parts of Khulna, a substantial buffalo population is also found, due to the availability of natural grazing facilities. Other districts, such as Rajshahi and Rangpur, have limited buffalo populations, primarily in intensive farming practices. Several factors, including season, age of calving, calving interval, dry period, herd population, agroecological region, and production system, are found to have a significant influence on buffalo milk production. A study on buffalo milk production across different agroecological zones in Bangladesh found that the highest average milk yield over 300 days was recorded in the riverine region of Lalpur (1,076 kg), while the lowest was observed in the coastal area of Bhola (592 kg) (Omar et al. 2024). However, the lower per-animal milk yield could also be a reflection of poor milk-yielding genetics. There are no defined buffalo breeds in Bangladesh; different indigenous river and swamp types and some imported breeds, including Murrah, Nili Ravi and their crosses with indigenous are distributed throughout the country (Sohel 2015; Hamid et al. 2017) (Fig. 3). Most of the areas comprise indigenous breeds, which are well-suited to the local climate and management systems but have lower milk yield (Samad 2020). To enhance productivity, in some areas, a few farmers have adopted crossbred buffalo, which combine traits of indigenous and high-yielding breeds and are known for comparatively higher milk yields per animal (e.g., Murrah, Nili-Ravi). It is therefore evident by the fact that buffalo in commercial farms produce 1.9 times more milk per day than the small-scale farm (Chanda et al. 2021).

### Buffalo rearing system

Primarily, there are five buffalo rearing systems in the country, such as free-range or bathan, semi-free-range or semi-bathan, intensive, semi-intensive, and household. A brief overview of the buffalo rearing systems and the physical characteristics of the buffalo breeds is given in **(**Table 1). In Bangladesh, the intensive buffalo farms have recently been rising, and there are a few government and private-owned buffalo farms, each with a distinctive purpose given in Table 2.

**Table 1:** Comparative features, including potential opportunities and challenges of different rearing systems, are outlined below (Samad 2020; Sagor et al. 2024)

Key features	Free-range or Bathan	Semi-free-range or semi-bathan	Intensive	Semi intensive	Household
Suitable area	Coastal region (Bhola, Noakhali, Lakshmipur and Patuakhali districts)	Coastal and semi-coastal region (Rice harvesting season)	Noakhali, Mymensingh, and Natore districts	Plain and marshy land where grazing land is limited (Rajshahi district)	Marshy land, heavy draft area (Tangail, Jamalpur, Bogura, Sirajganj, Pabna districts)
Herd size (Average buffalo heads)	50–200	4–15	20–200	4–15	1–3
Infrastructure	Under the open sky at night (91%), under the tree (5%) and few get shelter at night (3%)	Kept in household during rice cultivation and the remaining period kept free in common land, islands, and wetlands.	Reared in shed and stall-fed	Combination of free-range and household systems mostly kept in the yard or near the house at night without any roof	Reared at the backyard of the houses
Feed source	Extensive vegetation for grazing	Crop residues, straw, seasonal paddy field grasses	Cultivated fodder e.g., Napier, German, and Jumbo, rice straw and concentrate mixture.	Extensive vegetation in grazing land with no or little supplementation	6–7 h of grazing in the backyard with little or no feed supplementation.
Potential	Less feed requirements	Less feed requirements	Commercially profitable rearing system in terms of available resources	Sustainable rearing system in terms of limited resources	Management is comparatively easy in household families
Constraints	Limited fodder and grazing land, low productivity, lack of AI facilities	Limited feed and fodder sources, low productivity	Requires large investments and restricted movement limits the animal welfare	High price of feed and low milk price	Low production of milk and shortage of feed and fodder

**Table 2:** Comparative overview of notable intensive buffalo farms from government and non-government sectors (Seraj 2021; SAARC 2024)

Feature	Government Buffalo Breeding and Development Farm, Bagerhat	Government Buffalo Breeding and Development Farm, Tangail	Milk Vita Buffalo Farm, Takerhat	Milk Vita Buffalo Farm, Lakshmipur	Bangladesh Livestock Research Institute (BLRI) Buffalo Farm	SS Cattle Farm, Narayanganj	Lal Teer Livestock Development (BD) Limited	American Dairy Limited (ADL)
Ownership	Government-operated		Cooperative under Bangladesh Milk Producers Co-operative Union Ltd.		Government research institute	Privately owned	Private company	Private company
Location	Bagerhat District, Khulna Division	Tangail District, Dhaka Division	Madaripur District, Dhaka Division	Lakshmipur District, Chattogram Division	Savar, Dhaka Division	Narayanganj District, Dhaka Division	Bhaluka, Mymensingh District	Gazipur District, Dhaka Division
Breeds	Focus on Nili-Ravi and Murrah breeds		Murrah buffalo	Murrah buffalo	Indigenous and crossbred buffalo	Nili-Ravi, Murrah, Jafarabadi, Kundi, and pink buffalo	Mediterranean, Murrah, Nili-Ravi, and local varieties	Nili-Ravi and Murrah
Primary Objectives	Breeding and development of high-yielding dairy buffalo; supplying hybrid buffalo to farmers at low cost		Enhancing milk production through high-yielding buffalo breeds		Research and development in livestock breeding, nutrition, and health	Commercial farming with a focus on organic rearing and breed diversification	Genetic improvement of dairy and beef buffalo; production and distribution of high-quality semen; training AI insemination service providers	Genetic improvement of cattle and buffalo; dairy production; research and development
Facilities	Extensive grazing fields, breeding facilities, veterinary services		Breeding facilities, milk collection, and processing units		Research laboratories, breeding facilities, experimental farms	Land (approximately 3.2 acres) with large sheds for various livestock	Nucleus genetic farm, bull station for frozen semen production, training center for AI service providers	Dairy breeding herd, breeding bull station, milk processing plant, bull station, AI lab
Challenges	Low performance and continuous efforts to promote Murrah breed for higher yield		Maintaining genetic diversity; adapting imported breeds to local conditions Not specified		Ensuring the availability of quality breeding stock	High maintenance costs	Ensuring the availability of quality breeding stock	High operational costs; need for advanced technology adoption

### Buffalo diseases

**Fig. 4:**
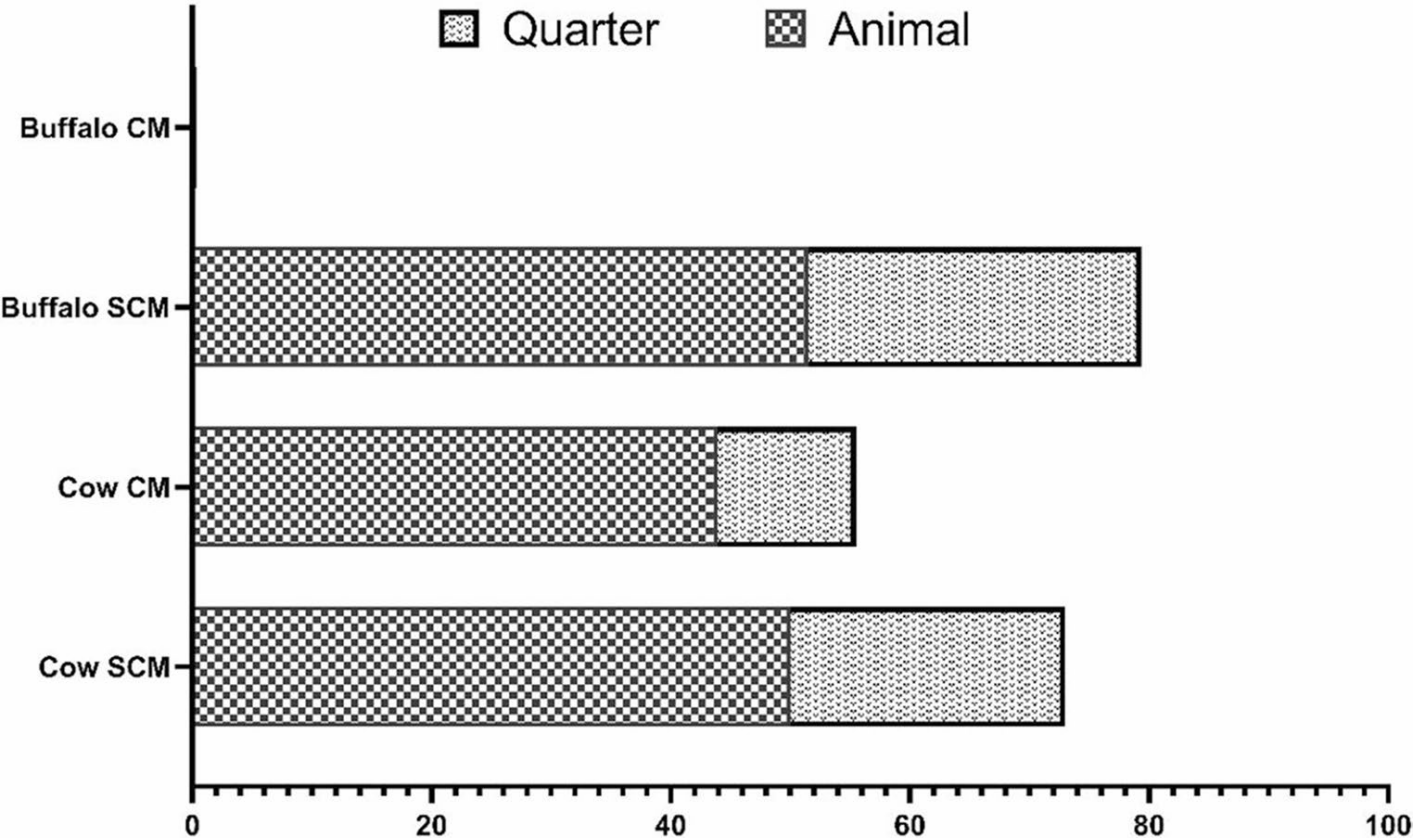
Overview of occurrences of clinical and subclinical mastitis in cattle and buffalo. The figure was adapted from the data provided in two previous studies (Singha et al. 2021a, 2021b, 2023a, 2023b)

Buffalo are known to be more resistant to many diseases than cattle due to their genetic traits and adaptive responses, which help them to survive in adverse environmental conditions (Martínez-Burnes et al. 2024). In Bangladesh, the most prevalent diseases include gastrointestinal parasitic infections and helminthiasis. A study on gastrointestinal parasitic infestations in buffalo highlighted that approximately 64% of buffalo were infected with one or more types of gastrointestinal parasitic species, which may result from poor hygiene and inadequate deworming practices, leading to severe impacts on growth and productivity (Islam et al. 2016a). Among parasitic diseases, hydatidosis also poses a significant concern, with infection rates exceeding 40% (Islam 1982). Hemorrhagic septicemia, a bacterial disease with a high mortality rate, poses a considerable threat, highlighting the urgent need for systematic vaccination programs. Subclinical mastitis (SCM) a common issue that hinder milk production, often due to poor udder hygiene and a lack of preventive measures. Several studies were conducted on mastitis and the reported prevalence of clinical mastitis (CM) in water buffalo was negligible compared to cows (Singha et al. 2021a, 2021b, 2023a, 2023b) (Fig. 4). Apart from the udder diseases, calf pneumonia, primarily affecting young buffalo, is frequently associated with poor housing and environmental management. Bacterial enteritis and other digestive diseases also underline the importance of maintaining proper feeding and hygiene standards. In buffalo calves, enteritis due to parasitic and protozoal infestations is a major concern, among which toxocariasis is the most prevalent, affecting over 50% of the population. Among the reproductive disorders in buffalo, uterine prolapse and repeat breeding are common, often linked to nutritional deficiencies and improper breeding practices, which reflect the limited availability of high-quality feed and lack of scientific breeding programs. Strengthening government and private sector initiatives, along with international collaboration, can significantly enhance disease prevention and improve the overall productivity of buffalo farming in Bangladesh.

### Buffalo product, market potential, and food safety

Buffalo milk is popular for its high nutritional value, which is enriched with higher fat, protein, and total solids compared to cow milk, making it ideal for dairy products such as yogurt, butter, ghee, and cheese. Fermented milk or yogurt from Bhola and Noakhali districts is quite in demand. Buffalo milk is mainly popularized in Mymensingh, Bhola, Noakhali, Natore, and Pabna districts due to the presence of high fat and chhana (acid curd of milk) that are produced during the processing. Despite this, buffalo milk production in Bangladesh accounts for only 0.04% of the global total, indicating substantial room for improving the per animal milk yield through better farm management (Chakravarty 2013).

**Fig. 5:**
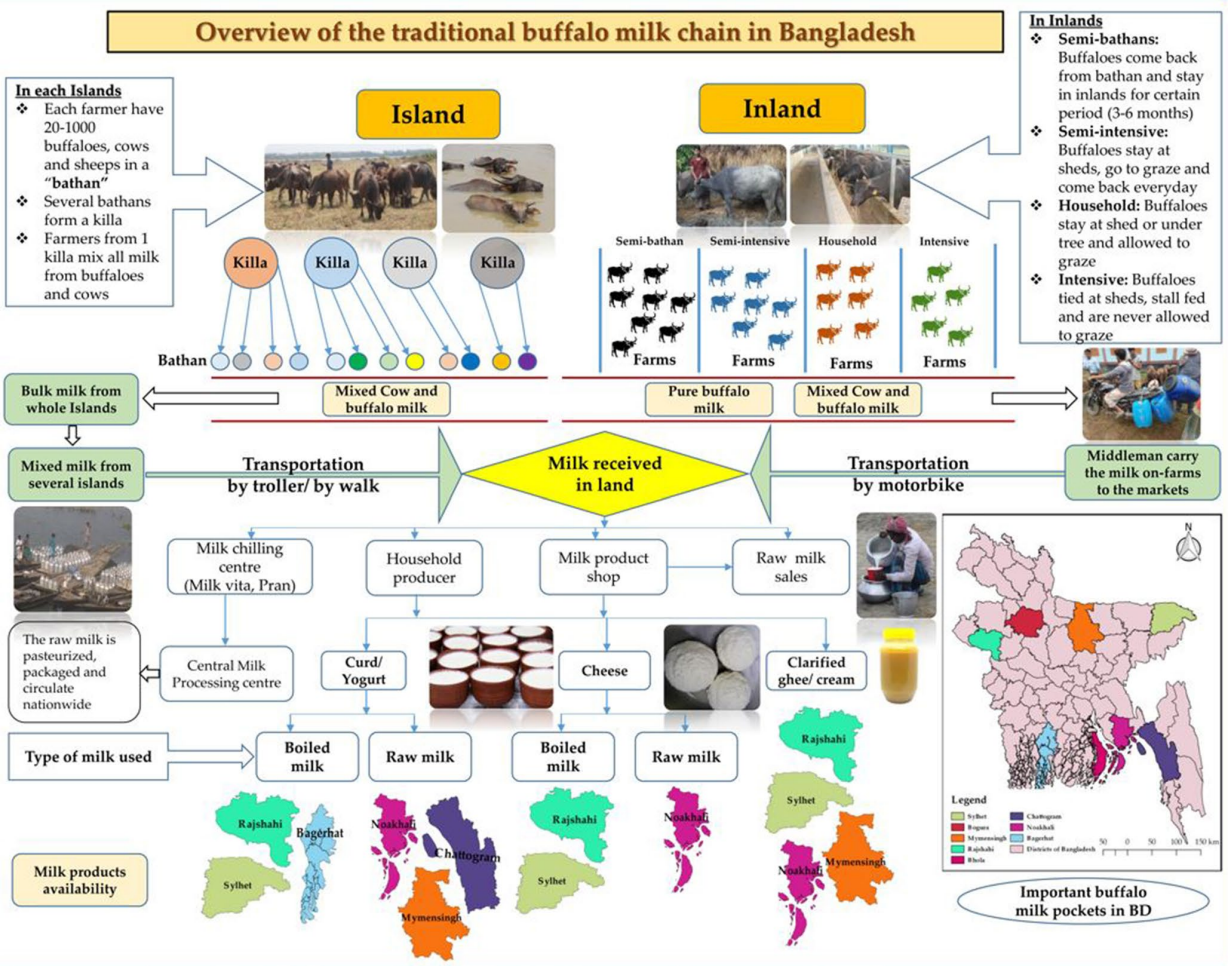
Schematic flow of the water buffalo milk supply chain in Bangladesh (Singha et al. 2023a, 2023b)

The existing informal buffalo milk supply chain is complex (Fig. 5), and the absence of a dedicated, organized buffalo product chain, similar to the one for cattle in Bangladesh, has reduced the marketplace for buffalo products, compromised the profit margin for farmers, and led to higher prices for consumers. A separate product value chain for buffalo products may help ensure the authenticity and availability of these products to consumers.

In Bangladesh, buffalo milk trading operates at various levels, including middlemen, milk collection centers, and milk product shops. The informal buffalo milk supply chain begins with milk producers at the production level, followed by vendors at milk collection centers and commercial milk processors. Milk is primarily collected from remote areas by middlemen and milk product producers, who then supply it to dairy product manufacturing companies or sell it in local markets. Traditionally, buffalo milk is processed into products like yogurt and cheese using raw or boiled milk, often without pasteurization (Singha et al. 2023a, 2023b). Typically, buffalo farms are situated far from milk processing facilities, with minimal adherence to hygienic practices.

### Udder health and milk quality

**Fig. 6:**
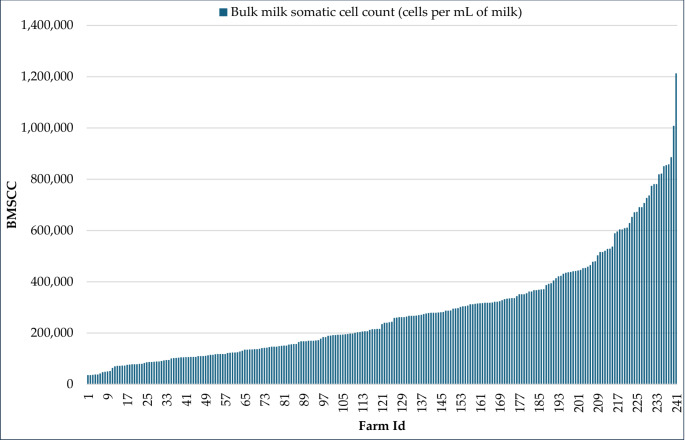
Farm level bulk milk somatic cell counts on 241 buffalo water buffalo farms in a cross-sectional study conducted in 7 districts in Bangladesh. The data was adapted from a previous study (Singha et al. 2023a, 2023b)

Bulk milk somatic cell count (BMSCC) and total bacterial count (TBC) are key indicators representing the udder health and milk quality in water buffalo. High levels of somatic cells and bacterial contamination, e.g., Staphylococci, Enterobacteriaceae, were observed in milk, reflecting suboptimal hygiene practices during milking and handling. Research findings on buffalo milk quality reported that 30% of farms had BMSCC exceeding the threshold of 400,000 cells per mL according to the European Community for Bovine milk (Costa et al. 2020) (Fig. 6) where only 12.5% of farms performed pre- and post-dipping practices during milking. Intensive buffalo farming systems had higher BMSCC levels compared to semi-intensive systems, indicating stress-related issues such as discomfort and limited movement. Seasonal stress during spring also increased BMSCC levels compared to winter (Singha et al. 2023a, 2023b). The Total Bacterial Count (TBC) increased along the milk chain, from 5.2 log10 CFU per mL at the farm level to 7.5 log10 CFU per mL in milk products, likely due to extended transportation times, labor-intensive handling processes, and inadequate milk cooling infrastructure. This leads to contamination by various foodborne pathogens, such as *Staphylococcus aureus*, *Escherichia coli*, and other enteropathogenic organisms, both on the farm and during the supply chain, since no cold chain is available during transportation (Singha et al. 2023a, 2023b, 2024a, 2024b) (Tables 3, 4 and 5). Therefore, improving udder health and ensuring hygienic practices throughout the buffalo milk value chain are crucial to enhance the safety and quality of buffalo milk.

**Table 3:** Bacterial contamination and prevalence of different zoonotic pathogens from the milk and milk product samples (*N* = 143) collected from four different nodes of the Buffalo milk chain in Bangladesh. The table was adapted from the data provided in a previous study (Singha et al. 2024a, 2024b)

Pathogen name	Type of samples (%)			
	Bulk milk	Middleman	Milk collection center	Milk products
*Staphylococcus. aureus*	8.8	13.5	21.6	31.4
*Escherichia. coli*	97.1	81.1	89.2	88.6
*STEC E. coli*	52.9	29.7	48.6	74.3
*Listeria monocytogenes*	64.7	43.2	5.4	14.3
*Yersinia enterocolitica*	79.4	0.0	18.9	2.9
*Salmonella* spp	2.9	0.0	13.5	2.9
*Campylobacter jejuni*	0.0	0.0	0.0	0.0

**Table 4:** Descriptive statistics for bulk milk somatic cell count (BMSCC), total bacterial count (TBC), total Staphylococcus aureus count (TSA), total non-aureus staphylococcus count (TNAS), and total Enterobacteriaceae count (TEC) at different nodes of informal Buffalo milk value chain. The table was adapted from the data provided in a previous study (Singha et al. 2023a, 2023b)

Parameters^1^	Nodes of milk value chain	Min-Max	Median	Mean ± SE (Mean)
BMSCC	Farm	4.6 to 6.1	5.4	5.4 ± 0.03
TBC	Farm	1.9 to 7.3	5.2	5.2 ± 0.08
	Middleman	3.4 to 8.3	5.9	5.9 ± 0.10
	Milk collection centre	3.6 to 9.9	6.7	6.6 ± 0.10
	Milk products	3.6 to 9.9	7.5	7.5 ± 0.19
TSA	Farm	2.9 to 3.9	3.1	3.2 ± 0.09
	Middleman	2.9 to 5.3	3.4	3.6 ± 0.17
	Milk collection centre	2.9 to 5.7	3.6	3.6 ± 0.16
	Milk products	2.9 to 5.9	3.8	3.9 ± 0.55
TNAS	Farm	2.9 to 6.7	4.2	4.4 ± 0.09
	Middleman	2.9 to 7.9	4.9	4.9 ± 0.11
	Milk collection centre	3.3 to 8.4	5.5	5.4 ± 0.11
	Milk products	3.6 to 7.7	5.7	5.8 ± 0.24
TEC	Farm	1.9 to 5.7	2.8	2.9 ± 0.12
	Middleman	1.9 to 7.4	3.8	4.1 ± 0.19
	Milk collection centre	1.9 to 7.0	4.1	4.2 ± 0.15
	Milk products	2.3 to 8.4	4.8	4.6 ± 0.27

^1^*BMSCC* Bulk milk somatic cell count per mL of milk, *TBC* Total bacterial count per mL of milk, *TSA* Total staphylococcus aureus count per mL of milk, *TNAS* Total non-aureus Staphylococci per mL of milk, *TEC* Total Enterobacteriaceae count per mL of milk

**Table 5:** Pathogen specific quarter level prevalence of organisms in Buffalo cows in different districts of Bangladesh

Isolated bacteria	No of quarters tested	95% CI	Number of affected quarters (%)	Ref
*Staphylococcus aureus*	188	0.33–0.48	76 (40.4)	(Hoque et al. 2022)
*S. aureus*	1364	0.20 − 0.04	39 (2.8)	(Singha et al. 2024a, 2024b)
*Streptococcus spp.*,	1364	0.003–0.12	10 (0.7)	(Singha et al. 2024a, 2024b)
*Streptococcus agalactiae*	188	0.03–0.10	11 (5.9)	(Hoque et al. 2022)
*Streptococcus uberis*	188	0.02–0.08	7 (3.7)	(Hoque et al. 2022)
*Streptococcus dysgalactiae*	188	0.01–0.07	6 (3.2)	(Hoque et al. 2022)
*Escherichia coli*	188	0.04–0.12	13 (6.9)	(Hoque et al. 2022)
*Klebsiella spp.*	188	0.02–0.08	7 (3.7)	(Hoque et al. 2022)
*Mammalicoccus*	1364	0.05–0.08	85 (6.2)	(Singha et al. 2024a, 2024b)
NAS*	1364	0.23–0.28	354 (25.8)	(Singha et al. 2024a, 2024b)

*NAS = Non-aureus Staphylococcus

A nationwide cross-sectional study in Bangladesh reported that overall SCM prevalence was high at 27.9% at the quarter-level and 51.5% at the buffalo-level. The geometric mean of BMSCC was 217,000 cells/mL of milk, which is low on average; however, some farms could improve substantially. An earlier study showed that the prevalence of intramammary infection (IMI) in water buffalo was high and varied between farms (Table 5). Buffalo herds with poor milking hygiene had a high prevalence of IMI by any pathogen or by non-aureus staphylococci and mammallicocci (NASM). Poor cleanliness of the hind quarters and asymmetrical udders were associated with an IMI by NASM and by any bacteria, respectively. However, udder asymmetry is likely linked to the persistence of IMI-causing pathogens from previous clinical infections, which may be induced by scar tissue formation and subsequent shrinkage of the udder gland. These pathogens can be transmitted and leading to the leading to the emergence of new IMI cases. Antibiogram studies in buffalo milk showed that *S. aureus* and *E. coli* indicate high resistance to ampicillin and tetracycline (Biswas et al. 2020) (Table 6). A few studies have identified antimicrobial resistance (AMR) genes in buffalo milk, indicating the presence of multidrug-resistant strains. In *E. coli* isolates, a variety of resistance genes were detected, including *stx1* and *stx2* (Shiga toxin genes), *aac(3)-iv* (aminoglycoside resistance gene), *tetA* (tetracycline resistance gene), *sul1* (sulfonamide resistance gene), *strA* (streptomycin resistance gene), and several extended-spectrum beta-lactamase (ESBL) genes such as *blaCTX-M* group 1, 2, and 9 (Gupta et al. 2018). Similarly, *aac(3)-iv*, *tetA*, *sul1*, *strA*, and multiple *blaCTX*-M group genes were also found in *Klebsiella* spp. isolates, highlighting the potential risk of multidrug resistance (Chowdhury et al. 2025). There is no clear evidence that antibiotic use is higher in buffalo compared to cattle; in fact, it is likely lower. Interestingly, resistance levels in buffalo to commonly used antimicrobials appear slightly lower than those reported in cases of intramammary infections (IMI) in cattle in Bangladesh (Table 6). However, fewer studies have been conducted on AMR in buffalo compared to cattle, which may lead to an underrepresentation of the resistance spectrum in buffalo. For a better understanding of resistance dynamics and to guide appropriate antimicrobial use in the sector, there is a need for more comprehensive surveillance and extensive studies.

**Table 6:** Antimicrobial resistance (AMR) profiles and associated AMR genes of bacterial isolates from cattle and Buffalo (Salauddin et al. 2019; Singha et al. 2021a, 2021b; bag et al. 2022; Hoque et al. 2022; Islam et al. 2025)

Organism tested	Antimicrobial Resistance %*															
	*P*		AMP		AMX		GEN		CIP		AZM		DOX		TE	
	Cattle	Buffalo	Cattle	Buffalo	Cattle	Buffalo	Cattle	Buffalo	Cattle	Buffalo	Cattle	Buffalo	Cattle	Buffalo	Cattle	Buffalo
*Staohylococcus aureus*	100	100	85	92	75	-	50	42		59	57	0	-	90	66	80.7
*Enterococcus fecalis*	0	-	0	-	0	-	0	-	0	-	40	-	-	-	40	-
*Non-aureus Staphylococci*	69	36	-	-	-	-	66	-	-	-	-	-	-	-	43	
*Escherichia coli*	-	-	89	55	94	-	36	92–100	5.3	92–100	88	0			90	90
*Klebsiella* spp	-	-	-	90–100	100	-	0	10–30	0	-	0	-	-	-	100	60–80

* Antimicrobial resistance percentage was reported based on the findings from the published original research articles in Bangladesh. Penicillin (P), Ampicillin (AMP), Amoxicillin (AMX), Gentamicin (GEN), Ciprofloxacin (CIP), Azithromycin (AZM), Doxycycline (DOX), and Tetracycline (TE)

### Prospects and constraints of buffalo farming

**Fig. 7:**
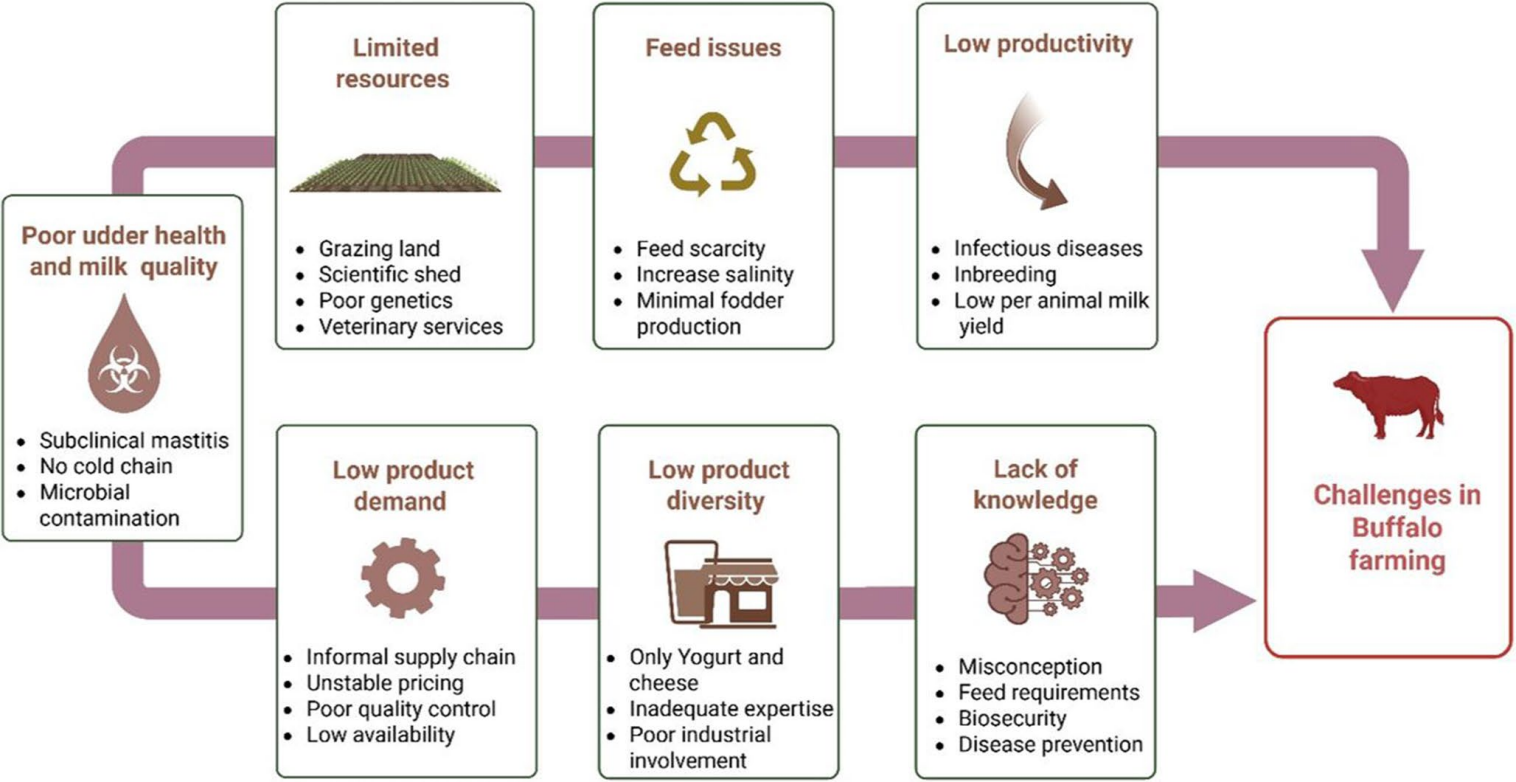
Overview of the key challenges in water buffalo farming, highlighting issues related to resources, feed, productivity, product demand, diversity, and knowledge gaps. The graphics was created using BioRender

Buffalo has the potential to address the growing demand for protein in the country. With an increasing population, the domestic and international markets for high-quality buffalo products offer significant opportunities for farmers. Buffalo are well-adapted to the local climatic conditions, thriving in flood-prone coastal and riverine areas, which makes them ideal livestock species for utilizing lands with minimal agricultural production. Their resilience to harsh environments and ability to efficiently convert low-quality feed into valuable products further underscore their importance in sustainable farming systems. However, buffalo farming in Bangladesh faces several constraints that hinder its growth and productivity (Fig. 7). The primary challenge is the low milk yield potential of the indigenous buffalo breed (1–3 L per day milk yield), the absence of organized breeding infrastructure and the lack of a genetic improvement programme resulting in lower milk compared to riverine breeds (Omar et al. 2024). Feed and fodder scarcity is another significant issue, as farmers struggle to provide adequate and quality nutrition year-round. Poor management practices, including a lack of technical knowledge among farmers and inadequate housing, further limit productivity. Health-related issues, such as disease prevalence and limited access to veterinary services, exacerbate the problem. The traditional farming systems, including extensive or bathan farming, often fail to provide the necessary preventive measures (vaccination, deworming) and nutrition required to maintain buffalo health. Additionally, market-related challenges include poorly organized markets for buffalo products and the absence of separate value chains hinder the profitability of the buffalo products. Policy gaps, such as the lack of effective buffalo-development programs and insufficient extension and research work, further lack of collaboration and coordination among different stakeholders related to buffalo hamper the long-term development of the buffalo sector (Saadullah 2012). Altogether, these problems lead to low productivity per animal, but also to a lack of enthusiasm for farmers to shift from cow to buffalo production. To resolve these issues, there is a need for education of farmers, better organization of the milk chain and better buffalo genetics. Addressing these constraints through strategic interventions, we expect that buffalo production increases substantially both in milk yield per animal and per farms, and we expect better product quality and food safety.

### Previous research overview on water buffalo in Bangladesh

Though buffalo are the second most important livestock species in Bangladesh, there are still limited documented research available on some key areas, including their farming practices. Various studies have been conducted focusing on different aspects of buffalo farming, such as reproduction, production traits, disease patterns, and management systems. However, many key areas remain underexplored, including breed development, fodder improvement, and enhancing productive and reproductive performance through genetic advancements. More collaborative research is crucial to address these gaps and improve the productivity, sustainability, and profitability of buffalo farming practices in Bangladesh **(**Table 7**)**.

**Table 7:** Overview of key research areas in buffalo farming, summarizing current findings and outlining the potential areas for future research

Research areas	Research findings	Future research scope
Demography and Management	• Productive and reproductive characteristics of different buffalo breeds (Rashid et al. 2019) • Morphological characterization of buffalo available in the coastal areas (Saha et al. 2018) • Socio-economic status of buffalo farmers (Sarkar et al. 2013; Haque et al. 2020; Hossain et al. 2021) • Differential management practices of buffalo farming in different areas (Rahman et al. 2019a, 2019b; Kabir et al. 2020; Chanda et al. 2021; Hossain et al. 2021) • Differential production status of buffalo in small-scale and commercial farming systems (Chanda et al. 2021) • Effect of supplementation of high and low energy diets in production traits (Siddiki et al. 2021)	• Establishing region-specific fodder cultivation • Understanding the effect of climate resilient housing system on health and production
Genetic diversities	• Genetic diversity of Bangladeshi buffalo (Faruque 2007)	• Comprehensive genetic profiling of indigenous water buffalo • Development of climate-resilient buffalo breeds • Genetic variability of buffalo in different management system
Reproduction	• Seasonal variation of semen characteristics (Sharma et al. 2018) • Different methods to enhance the pregnancy rate in buffalo (Paul et al. 2025) • Evaluate insemination time and pregnancy rate (Hamid 2019) • Effect of Showering on semen quality (Hoque et al. 2018) • Effect of parity on reproductive and productive status (Fakruzzaman et al. 2020; Khaton et al. 2020) • Estrus synchronization using PGF2α in native buffalo (Roy et al. 2022)	• AI techniques and semen preservation methods to improve conception rate • Effect and factors associated with inbreeding in buffalo(Saadullah 2012)
Production	• Overview of milk production performances of buffalo (Faruque et al. 2019) • Factors affecting milk production traits in indigenous buffalo (Omar et al. 2024) • Blood biochemical parameters of buffalo reared in the high salinity area of Bangladesh (Runa et al. 2022)	• Approaches to mitigate the low production performance of indigenous buffalo
Buffalo product	• Nutritional profiling of buffalo products (Asif et al. 2021; Md Asif et al. 2022) • Genetic polymorphism and their association with milk yield in river buffalo • Nutritional and microbiome diversity of buffalo products (Islam et al. 2021) • Detection of alteration in buffalo products (Afifa Khatun et al. 2021)	• Potential for developing buffalo-based dairy products.
Market chain	• Current marketing approaches of buffalo milk (Rahman et al. 2019a, 2019b) • Demographic characteristics of buffalo traders (Habib et al. 2021a, 2021b)	• Challenges and opportunities in establishing a formal and traditional buffalo product value chain. • Market potential for value-added buffalo milk products • Supply chain efficiency in buffalo milk production
Milk quality	• Factors influencing somatic cell counts and bacterial contamination in water buffalo milk (Singha et al. 2023a, 2023b) • Comparative analysis of milk components of buffalo and cattle • Prevalence of food-borne bacteria in the buffalo value chain (Islam et al. 2018; Singha et al. 2024a, 2024b)	• Milk quality parameters and associated factors of buffalo colostrum
Udder health	• Intramammary infection in buffalo (Singha et al. 2024a, 2024b) • Prevalence and risk factors of sub-clinical mastitis (SCM) (Singha et al. 2023a, 2023b) (Hoque et al. 2015) (Biswas et al. 2020) • Risk factors for intramammary infection in water buffalo (Singha et al. 2024a, 2024b) • Etiology associated with SCM (Singha et al. 2021a, 2021b) • Antibiogram and virulence profiling of MDR *S. aureus* in riverine buffalo (Hoque et al. 2022)	• Comparative Analysis of milk quality in buffalo vs. cow milk chains • AMR and antibiotic residue in buffalo milk and the traditional value chain • Virulence gene profiling of pathogenic organism available in buffalo milk and milk chain • Resistome pattern of buffalo CM and SCM microbiome
Disease and treatment	• Epidemiological status of buffalo diseases (Islam et al. 2016a, 2016b; Bhuiyan et al. 2019) • Perception of large-animal farmers towards antimicrobial use, resistance, and residues (Hossain et al. 2022) • Prevalence of FMD & HS in buffalo (Ujjal et al. 2018) • Prevalence of gastrointestinal parasites (Mamun et al. 2011; Ara et al. 2021; Biswas et al. 2021) • Prevalence of brucellosis (Rahman et al. 2011) (Islam et al. 2013) • Prevalence of hydatidosis in buffalo (Islam 1982) • Efficient anthelminthics against toxocariosis in buffalo calves (Biswas et al. 2022) • Genomic sequence of *Pasteurella multocida* responsible for mortality of bovines (Sarker et al. 2018) • Molecular and genetic variability of *Toxocara vitularum* infecting buffalo calves (Biswas et al. 2024) • Time-space cluster and risk factors of fasciolosis and FMD in domestic animals (Rahman et al. 2017; Ara et al. 2021) • Molecular detection of Cryptosporidium in Buffalo (Mahen et al. 2024) • Genetic characterization of Shiga toxin-producing *E coli* from livestock (Islam et al. 2008, 2010) • Genomic sequences of buffalo corona virus (Ahsan et al. 2014)	• Evaluation of the efficiency of available vaccines • Emerging infectious diseases of buffalo in face of the climate change • Efficiency of anthelmintics in different buffalo management system by in vitro egg hatch assay (EHA) test
Potential and constraints	• Potentiality and constraints of buffalo production (Saadullah 2012) • Economic benefit of small-scale dairy buffalo farming (Islam et al. 2017; Habib et al. 2021a, 2021b)	• Prospects of climate adaptable buffalo farming

### Key scientific research achievements on the water buffalo

Besides the government, various national and international organizations, through non-governmental development partners in Bangladesh, both independently and in collaboration, have made significant strides in buffalo development. Their efforts have mainly focused on activities such as, breed development providing high yielding bulls to the farmers, initiating widespread dissemination of artificial insemination, establishing semen banks and germplasm, studying physiological responses of buffalo to heat stress, safety assessments of dairy products through surveillance of zoonotic pathogens, and investigating antibiotic use in dairy production. Additionally, capacity-building efforts, such as training on eco-friendly buffalo rearing, safe dairy processing, livestock waste management, and organizing vaccination and deworming campaigns, are crucial for fostering sustainable practices and enhancing the country’s buffalo farming environment.

Overall recommendations to improve buffalo production in Bangladesh.

The key recommendations that can be drawn from the existing efforts to mitigate the current constraints moving towards a sustainable and profitable dairy buffalo production in the country are listed below,

- Enhance genetics with high-yield breeds and selective breeding, improving the AI facilities.
- Reserve grazing lands and build protective infrastructure.
- Focus on dry cow management and monitoring of udder health and SCM.
- Improving farm management practices by addressing the contamination risks along the milk chain.
- Enforce a higher farm udder health hygiene standard with a gradual reduction in BMSCC, setting a target of ≤ 200,000 cells/mL.
- Implement milk quality monitoring across dairy zones and set standard limits for bacterial counts to ensure milk hygiene and improvement of udder health.
- Develop a national buffalo dairy policy for milk safety and quality.
- Establish a cooperative-based buffalo product market value chain through a collaborative initiative between the government and private sectors for enhancing fair pricing and product demand.
- Strengthen the biosecurity and hygienic practices both in farm environment and farm personnel to reduce the risk of disease transmission.
- Availability of affordable, high-quality feed and fresh water supply to enhance the production efficiency.
- Train farmers on housing, management, and modern husbandry practices.
- Ensure sufficient disease surveillance and veterinary care in the buffalo herd health approach.

## Conclusions

Water buffalo production in Bangladesh holds substantial potential; however, it is hindered by key challenges, including low per-animal milk yield, high somatic cell counts, and widespread bacterial contamination, which indicate poor udder hygiene and suboptimal milking practices. The higher prevalence of important mastitis-causing and foodborne pathogens, along with notable resistance to beta-lactam antibiotics, underscores the urgent need for improved udder health management and the rational use of antibiotics. Specific interventions should focus on enhancing on-farm hygiene protocols, implementing routine mastitis screening, and promoting farmer training on milking hygiene. Moreover, targeted breeding programs, improved access to veterinary services, and supportive policy frameworks are crucial for addressing key challenges. Prioritizing these evidence-based strategies can significantly boost productivity, promote animal health, and support sustainable buffalo farming in Bangladesh.

## Electronic supplementary material

Below is the link to the electronic supplementary material.

Supplementary Material 1 (PDF 73.0 KB)

Supplementary Material 2 (PDF 301 KB)

## Data Availability

Data is provided within the manuscript or supplementary information file.
