# Supplementary material for: Water buffalo farming, udder health and its dairy production status in Bangladesh: Practices, challenges, and potentialities
Source: Vet Res Commun. 2025 Aug 28;49(5):292. doi: 10.1007/s11259-025-10823-8 (PMC12394335; doi:10.1007/s11259-025-10823-8)
Supplement: Supplementary file 1 — Supplementary Material 1 (PDF 73.0 KB) [file 11259_2025_10823_MOESM1_ESM.pdf]

**Supplementary file 1**

A list of the articles obtained through systematic search in the electronic database and with its eligibility status to be included in the final review
