# Supplementary material for: Water buffalo farming, udder health and its dairy production status in Bangladesh: Practices, challenges, and potentialities
Source: Vet Res Commun. 2025 Aug 28;49(5):292. doi: 10.1007/s11259-025-10823-8 (PMC12394335; doi:10.1007/s11259-025-10823-8)
Supplement: Supplementary file 2 — Supplementary Material 2 (PDF 301 KB) [file 11259_2025_10823_MOESM2_ESM.pdf]

Water buffalo farming, udder health and its dairy production status in Bangladesh: Practices, challenges, and potentialities

Tishita Sen Ape[1,2*], Shuvo Singha[2], Unusing Marma[1,2], Hasnat Jahan Rumi[1,2], Sirajul Islam Sagor[1,2], Antonella Chiariotti[3], Vittoria Lucia Barile[4], Ylva Persson[2,5,6], Md. Mizanur Rahman[1,2]

[1] Chattogram Veterinary and Animal Sciences University, Department of Medicine and Surgery, Chattogram, Bangladesh

[2] Udder Health Bangladesh, Chattogram, Bangladesh

[3] Council for Agricultural Research and Economics (CREA), Research Center for Animal Production and Aquaculture, Monterotondo, Italy

[4] Research Centre for Animal Production and Aquaculture, Consiglio per la Ricerca in Agricoltura e l'Analisi dell'Economia Agraria (CREA), Monterotondo, Italy

[5] The Swedish University of Agricultural Sciences, Department of Animal Biosciences, Uppsala, Sweden

[6] Swedish Veterinary Agency, Uppsala, Sweden

*****Correspondence: Tishita Sen Ape; Present address: Chattogram Veterinary and Animal Sciences University, Department of Medicine and Surgery, Chattogram, Bangladesh; Orchid Id: https://orcid.org/0009-0003-5387-7930, E-mail: tishitasen@student.cvasu.ac.bd; Tel: +880 1857531933

SL	First Author	Title	Acceptance/Rejection	Rejection reason	Relevant topic	Publication Year	Journal/Book	DOI
1	Paul AK	Intra-Vaginal Bio-Stimulation and Clitoral Massage to Enhance Pregnancy Rates in Water Buffalo in Coastal Bangladesh	Accepted		Reproduction	2025	Animals (Basel)	10.3390/ani15040597
2	Biswas H	Molecular characterization and genetic variability of Toxocara vitulorum from naturally infected buffalo calves for the first time in Bangladesh	Accepted		Disease	2024	Parasitology	10.1017/S0031182024000842
3	Singha S	Foodborne bacteria in milk and milk products along the water buffalo milk chain in Bangladesh	Accepted		Milk quality	2024	Sci Rep	10.1038/s41598-024-67705-3
4	Mou MA	Detection of Polymorphisms in FASN, DGAT1, and PPARGC1A Genes and Their Association with Milk Yield and Composition Traits in River Buffalo of Bangladesh	Accepted		Production	2024	Animals (Basel)	10.3390/ani14131945
5	Mahen MSK	Investigating the infection dynamics and molecular detection of Cryptosporidium in Buffaloes in Sylhet, Bangladesh	Accepted		Disease	2024	Vet Parasitol Reg Stud Reports	10.1016/j.vprsr.2024.101043
6	Omar AI	Factors Affecting the Milk Production Traits and Lactation Curve of the Indigenous River Buffalo Populations in Bangladesh	Accepted		Production	2024	Animals (Basel)	10.3390/ani14081248
7	Habiba MU	Draft genome sequence of Leuconostoc falkenbergense isolated from naturally fermented buffalo milk curd	Accepted		Buffalo products	2024	Microbiol Resour Announc	10.1128/mra.00148-24
8	Singha S	Pathogen group-specific risk factors for intramammary infection in water buffalo	Accepted		Udder Health	2024	PLoS One	10.1371/journal.pone.0299929
9	Habiba MU	Genomic insights into antibiotic resistance genes in Leuconostoc citreum strains isolated from artisanal buffalo milk curd in Bangladesh through whole-genome sequencing	Accepted		Buffalo products	2024	Microbiol Resour Announc	10.1128/mra.01289-23
10	Jolly YN	Heavy Metals Accumulation in Vegetables and Its Consequences on Human Health in the Areas Influenced by Industrial Activities	Rejected	Irrelevant		2024	Biol Trace Elem Res	10.1007/s12011-023-03923-6

SL	First Author	Title	Acceptance/Rejection	Rejection reason	Relevant topic	Publication Year	Journal/Book	DOI
11	Lu T	Risk mapping and risk factors analysis of r in livestock in Bangladesh using national-l passive surveillance data	Rejected	Not in buffalo		2023	Prev Vet Med	10.1016/j.prevetmed.2023.106016
12	Singha S	Factors influencing somatic cell counts anc bacterial contamination in unpasteurized m obtained from water buffalo in Bangladesh	Accepted		Milk quality	2023	Trop Anim Health Prod	10.1007/s11250-023-03644-x
13	Umair M	International manufacturing and trade in colistin, its implications in colistin resistan and One Health global policies: a microbiological, economic, and anthropolc study	Rejected	Irrelevant		2023	Lancet Microbe	10.1016/S2666-5247(22)00387-1
14	Singha S	The prevalence and risk factors of subclini mastitis in water buffalo (Bubalis bubalis) Bangladesh	Accepted		Udder Health	2023	Res Vet Sci	10.1016/j.rvsc.2023.03.004
15	Runa RA	Blood biochemical parameters of Murrah buffalo (Bubalus bubalis) reared in the hig salinity area of Bangladesh	Accepted		Production	2022	J Adv Vet Anim Res	10.5455/javar.2022.i643
16	Biswas H	Efficacy of clinically used anthelmintics ag toxocariasis of buffalo calves in Banglades	Accepted		Disease	2022	J Parasit Dis	10.1007/s12639-022-01522-1
17	Hoque MN	Antibiogram and virulence profiling reveal multidrug resistant Staphylococcus aureus the predominant aetiology of subclinical mastitis in riverine buffaloes	Accepted		Udder Health	2022	Vet Med Sci	10.1002/vms3.942
18	Khan A	Whole-Genome-Based Web Genomic Res for Water Buffalo (Bubalus bubalis)	Rejected	Not in Bangladesh		2022	Front Genet	10.3389/fgene.2022.809741
19	Hossain MT	A Survey on Knowledge, Attitude, and Practices of Large-Animal Farmers toward Antimicrobial Use, Resistance, and Residu Mymensingh Division of Bangladesh	Accepted		Disease	2022	Antibiotics (Basel)	10.3390/antibiotics11040442
20	Md Asif AH	Fatty acid and amino acid profiles of chees butter, and ghee made from buffalo milk	Accepted		Buffalo products	2022	J Adv Vet Anim Res	10.5455/javar.2022.i579

SL	First Author	Title	Acceptance/Rejection	Rejection reason	Relevant topic	Publication Year	Journal/Book	DOI
21	Afifa Khatun M	Detection of species adulteration in meat products and Mozzarella-type cheeses using duplex PCR of mitochondrial cyt b gene: A food safety concern in Bangladesh	Accepted		Buffalo products	2021	Food Chem (Oxf)	10.1016/j.fochms.2021.100017
22	Chowdhury EH	Peste des petits ruminants virus antibod in domestic large ruminants in Bangladesh	Rejected	Not in buffalo		2022	J Infect Dev Ctries	10.3855/jidc.15189
23	Rahman M	Epidemiology and molecular detection of Anaplasma spp. in goats from Chattogram district, Bangladesh	Rejected	Not in buffalo		2022	Vet Med Sci	10.1002/vms3.775
24	Biswas H	Prevalence and risk factors of Toxocara vitulorum infection in buffalo calves in coastal, northeastern and northwestern regions of Bangladesh	Accepted		Disease	2021	Vet Parasitol Reg Stud Reports	10.1016/j.vprsr.2021.100656
25	Islam SMR	Insights into the nutritional properties and microbiome diversity in sweet and sour yogurt manufactured in Bangladesh	Accepted		Buffalo products	2021	Sci Rep	10.1038/s41598-021-01852-9
26	Singh R	Current status and unique attributes of Indian Chilika buffalo for adaptation to brackish water ecology	Rejected	Not in Bangladesh		2021	Trop Anim Health Prod	10.1007/s11250-021-02973-z
27	Asif AHM	Variations in fatty acid and amino acid profiles of doi and rasomalai made from buffalo milk	Accepted		Buffalo products	2021	J Adv Vet Anim Res	10.5455/javar.2021.h541
28	Ara I	Prevalence and severity of gastro-intestinal parasites in buffalo calves at Sylhet division of Bangladesh	Accepted		Disease	2021	J Parasit Dis	10.1007/s12639-020-01339-w
29	Singha S	Occurrence and aetiology of subclinical mastitis in water buffalo in Bangladesh	Accepted		Udder Health	2021	J Dairy Res	10.1017/S0022029921000698
30	Roy BK	Scope and cost-effectiveness of fermented corn straw roughage-based buffalo fattening approach	Rejected	Not in Bangladesh		2021	J Adv Vet Anim Res	10.5455/javar.2021.h502

SL	First Author	Title	Acceptance/Rejection	Rejection reason	Relevant topic	Publication Year	Journal/Book	DOI
31	Banga-Mingo V	Whole gene analysis of a genotype G29P[6] human rotavirus strain identified in Central African Republic	Rejected	Not in Bangladesh		2021	BMC Res Notes	10.1186/s13104-021-05634-4
32	Sunita K	Buffalo Infection by Fasciola gigantica Transmitted by Radix acuminata in Uttar Pradesh, India: A Molecular Tool to Improve Snail Vector Epidemiology Assessments and Control Surveillance	Rejected	Not in Bangladesh		2021	Acta Parasitol	10.1007/s11686-021-00414-3
33	Youssef NA	Haplotype diversity in the mitochondrial genome of the Egyptian river buffalo (Bubalus bubalis)	Rejected	Not in Bangladesh		2021	Mitochondrial DNA B Resour	10.1080/23802359.2020.1852622
34	Sun T	Genetic diversity of mitochondrial cytochrome b gene in swamp buffalo	Rejected	Not in Bangladesh		2020	Anim Genet	10.1111/age.12997
35	Rahman AKMA	Foot-and-Mouth Disease Space-Time Clusters and Risk Factors in Cattle and Buffalo in Bangladesh	Accepted		Disease	2020	Pathogens	10.3390/pathogens9060423
36	Singh R	Mitochondrial sequence-based evolutionary analysis of riverine-swamp hybrid buffaloes of India indicates novel maternal differentiation and domestication patterns	Rejected	Not in Bangladesh		2020	Anim Genet	10.1111/age.12938
37	Harun-Or-Rashid M	Productive, reproductive, and estrus characteristics of different breeds of buffalo cows in Bangladesh	Accepted		Reproduction	2019	J Adv Vet Anim Res	10.5455/javar.2019.f382
38	Bhuiyan MAA	Animal-related injuries and fatalities: evidence from a large-scale population-based cross-sectional survey in rural Bangladesh	Accepted		Disease	2019	BMJ Open	10.1136/bmjopen-2019-030039
39	Sun T	Multiple domestication of swamp buffalo in China and South East Asia	Rejected	Not in Bangladesh		2020	J Anim Breed Genet	10.1111/jbg.12445
40	Mintoo AA	Draft genome of the river water buffalo	Rejected	Not in Bangladesh		2019	Ecol Evol	10.1002/ece3.4965

SL	First Author	Title	Acceptance/Rejection	Rejection reason	Relevant topic	Publication Year	Journal/Book	DOI
41	Sarker MSA	First Genome Sequence of Pasteurella multocida Type B Strain BAUTB2, a Major Pathogen Responsible for Mortality of Bovines in Bangladesh	Accepted		Disease	2018	Microbiol Resour Announc	10.1128/MRA.00901-18
42	Gupta MD	Occurrence of Escherichia coli carrying Shiga toxin-producing genes in buffaloes on smallholdings in Bangladesh	Accepted		Disease	2018	Vet World	10.14202/vetworld.2018.1454-1458
43	Thanh HD	Emergence of Human G2P[4] Rotaviruses in the Post-vaccination Era in South Korea: Footprints of Multiple Interspecies Re-assortment Events	Rejected	Not in Bangladesh		2018	Sci Rep	10.1038/s41598-018-24511-y
44	Faruk MO	Identification and molecular characterization of Echinococcus granulosus from domestic goat in Chittagong, Bangladesh	Rejected	Not in buffalo		2017	Trop Biomed	
45	Qiu Y	Emergence of an exotic strain of serotype O foot-and-mouth disease virus O/ME-SA/Ind-2001d in South-East Asia in 2015	Rejected	Not in Bangladesh		2018	Transbound Emerg Dis	10.1111/tbed.12687
46	Rahman AKMA	Fascioliasis risk factors and space-time clusters in domestic ruminants in Bangladesh	Accepted		Disease	2017	Parasit Vectors	10.1186/s13071-017-2168-7
47	Lau SK	First genome sequences of buffalo coronavirus from water buffaloes in Bangladesh	Accepted		Disease	2016	New Microbes New Infect	10.1016/j.nmni.2016.02.011
48	Borisevich SV	Buffalopox	Rejected	Not in Bangladesh		2016	Vopr Virusol	
49	Zhang Y	Strong and stable geographic differentiation of swamp buffalo maternal and paternal lineages indicates domestication in the China/Indochina border region	Rejected	Not in Bangladesh		2016	Mol Ecol	10.1111/mec.13518
50	Lederman E	Zoonotic parapoxviruses detected in symptomatic cattle in Bangladesh	Rejected	Not in buffalo		2014	BMC Res Notes	10.1186/1756-0500-7-816

SL	First Author	Title	Acceptance/Rejection	Rejection reason	Relevant topic	Publication Year	Journal/Book	DOI
51	Islam MA	Principal milk components in buffalo, holstein cross, indigenous cattle and red chittagong cattle from bangladesh	Accepted		Buffalo products	2014	Asian-Australas J Anim Sci	10.5713/ajas.2013.13586
52	Ahsan M	Handling and welfare of bovine livestock at local abattoirs in Bangladesh	Rejected	Not in buffalo		2014	J Appl Anim Welf Sci	10.1080/10888705.2014.905782
53	Islam MA	A review of Brucella seroprevalence among humans and animals in Bangladesh with special emphasis on epidemiology, risk factors and control opportunities	Accepted		Disease	2013	Vet Microbiol	10.1016/j.vetmic.2013.06.014
54	Haque N	An overview of Brucellosis	Rejected	Irrelevant		2011	Mymensingh Med J	
55	Islam MA	Occurrence and characterization of Shiga toxin-producing Escherichia coli in raw meat, raw milk, and street vended juices in Bangladesh	Accepted		Disease	2010	Foodborne Pathog Dis	10.1089/fpd.2010.0569
56	Islam MA	Prevalence and genetic characterization of shiga toxin-producing Escherichia coli isolates from slaughtered animals in Bangladesh	Accepted		Disease	2008	Appl Environ Microbiol	10.1128/AEM.00854-08
57	Ables GP	Sequence analysis of the NRAMP1 genes from different bovine and buffalo breeds	Rejected	Not in Bangladesh		2002	J Vet Med Sci	10.1292/jvms.64.1081
58	Nalin DR	A point survey of periurban and urban malaria in Karachi	Rejected	Not in Bangladesh		1985	J Trop Med Hyg	
59	Islam AWM	Hydatidosis in buffaloes in Bangladesh	Accepted		Disease	1982	Rev Sci Tech	10.20506/rst.1.2.71
